# Trends in mortality at Federal Medical Centre, Owo, Ondo State, Nigeria 2006-2014

**DOI:** 10.11604/pamj.supp.2019.32.1.13279

**Published:** 2019-01-25

**Authors:** Olayinka Stephen Ilesanmi, Abisola Oladimeji, Peter Adebayo Adewuyi, Patrick Mboya Nguku, Akin Oyemakinde, Olufunmilayo Ibitola Fawole, Uchenna Anebonam, Ahmed Abubakar

**Affiliations:** 1 Department of Community Health Federal Medical Centre, Owo, Ondo State, Nigeria; 2 Nigeria Field Epidemiology and Laboratory Training Programme, Abuja, Nigeria; 3 Department of Epidemiology and Medical Statistics, University of Ibadan, Nigeria

**Keywords:** Mortality, communicable disease, HIV, tuberculosis

## Abstract

**Introduction:**

vital events registration is not accurately done in Nigeria. Hence, mortality data is often not available. Health facility-based data can provide useful information on the trends in mortality of a population. This study describes the trend of deaths in a tertiary health facility.

**Methods:**

a retrospective review of the medical records of all patients that died in the Federal Medical Centre, Owo, Ondo State, Nigeria from 2006 to 2014 was conducted. Data extracted from the records included age, sex, primary cause of death and date of admission and death. Frequencies were done and the median age of male and female deaths were compared using Mann-Whitney U test.

**Results:**

a total of 1,326 deaths occurred in the hospital. The median age at death was 46 years (range: 0-110), median age was 49 years in males and 43 years in females (p = 0.025). One fifth (20.9%) of deaths was in infants. The median length of hospital stay was 4 days (range: 0-277). The highest proportion of death (16.4%) was in 2009 and on Wednesdays (15.9%). More deaths occurred in January (11.5%) and (10.3%) in February than in other months. The leading cause of communicable disease death in age 1 and above were HIV (15.2%) and TB (2.5%). Birth asphyxia (39.3%) and low birth weight (46%) were the commonest cause of death in infants.

**Conclusion:**

over the years the leading causes of mortality were preventable. Infant mortality remains a public health problem. Hospital mortality data could guide health decision making and interventions in Nigeria. Interventions to reduce death from communicable diseases and in infants are urgently recommended.

## Introduction

Registration of births, deaths, marriages and divorces are not accurately done in Nigeria. Due to the absence of these vital events registration mortality data is often unavailable [1]. Trends in mortality can be monitored using health facility based mortality data. Mortality data may include the number of deaths recorded by person, time, place and cause [2]. Cause-specific mortality data are important to monitor trends in mortality over time and are useful in designing strategies to reduce preventable death. Evidence based health decision and planning process involves consistent compilation of cause-specific and age-specific mortality data which is often unavailable in Nigeria. The hospital-based data, especially from general and well utilised hospitals may reflect the pattern of death in the community [3, 4]. Documentation on deaths is crucial to understand trends in mortality in the absence of reliable vital registration [5]. Data on deaths can improve informed health decision making and guide health planning to reduce mortality in many African countries. Information on local and national deaths by cause are essential to plan, manage and evaluate the performance of the health sector [6]. Hospital-based data can yield useful information to characterize mortality which has occurred and complement community-based data. It also contributes immensely in the evaluation of the quality of care provided in the health facility [4]. The information provided can guide identification of appropriate strategies to improve patient’s care and reduce or prevent recurrence of these deaths. This review aimed to determine the trends in mortality pattern at Federal Medical Centre, Owo, Ondo State, Nigeria from 2006 to 2014.

## Methods

**Study area:** the study was carried out at the Federal Medical Centre, a hospital located in Owo, Owo Local Government Area of Ondo State, Nigeria. The hospital serves as a referral health care facility to many of the states of the Federation because of its strategic location. Healthcare services are also provided to the people within its catchment areas which are Ondo, Kogi, Edo, Ekiti and Osun and other surrounding states. The centre provides postgraduate (residency) training in Medicine and Surgery. The centre has about 250 beds with a bed occupancy rate of about 70%. The hospital has about 1,200 staff including about 213 doctors and 300 nurses [7].

**Study design:** we conducted a cross-sectional study involving a retrospective review of records of patients who died at the Federal Medical Centre, Owo from January 2006 to December 2014. All patients who died in the hospital in all the departments and units were included in the review.

**Data collection:** information was extracted from the hospital mortality records. Variables available for review included, patients socio-demographic (age, sex, day, month, year, cause of death), admission and surgical processes, peri-operative and post-operative conditions and duration of hospital stay. The length of hospital stay was the total duration stayed in the hospital from the day of admission to the day of demise.

**Data analysis:** data entered by the hospital’s health information unit were already in MS Excel sheets. They were cleaned and analysed with SPSS version 21 [8]. Frequencies and proportions were done. The median age of death of male and female was compared using Mann-Whitney U test due to its skewness. The independent variables were socio-demographic variables including age and sex. Case specific variables were diagnosis, cause of death and length of hospital stay.

**Ethical considerations:** ethical approval for the study was obtained from the Health Research Ethics Committee of the Federal Medical Centre, Owo, Ondo State, Nigeria. Data collected was kept confidential on a password protected computer. Names and addresses were not included in the data extracted. Collected data could not be linked with deceased person.

## Results

**Figure 1:**
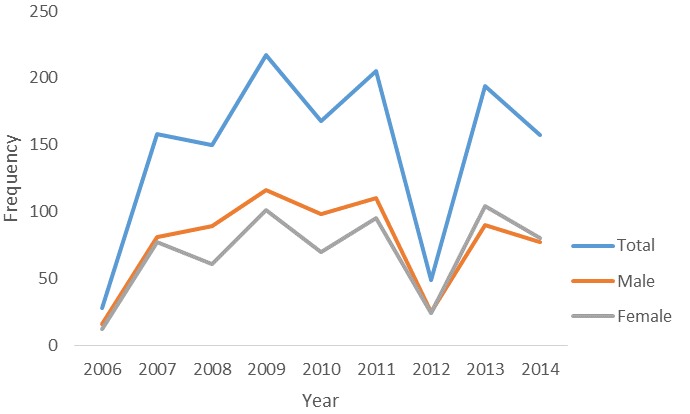
distribution of total death by sex, FMC Owo, 2006-2014

Over the nine-year period, 1,326 deaths occurred in the Centre. The median age at death was 46 years (range: 0-110 years). Figure 1 shows the age categories, 277 (20.9%) deaths were among children less than one year. In both sexes, aged 1 year and above median age of death was 46 years, (Inter Quartile Range [IQR]: 33 to 65 years). For males, the median age was 49 years (IQR: 35 to 65 years) and for females the median age was 43 years (IQR 31 to60 years), (p = 0.025). Males constituted 703 (53%). Of this subset, 335 (25.3%) were < 18 years, 56 (4.2%) and 272 (20.5%) were 65 years old and above. The sex and age group distribution are shown in Table 1.

**Table 1:** frequency and percentage of demises by selected characteristics Federal Medical Centre, Owo, 2006-2014

Variable	Frequency	%
**Sex**		
Female	623	47.0
Male	703	53.0
**Age group in years**		
<18	335	25.3
18-24	56	4.2
25-34	174	13.1
35-44	204	15.4
45-54	136	10.3
55-65	149	11.2
65+	272	20.5

**Figure 2:**
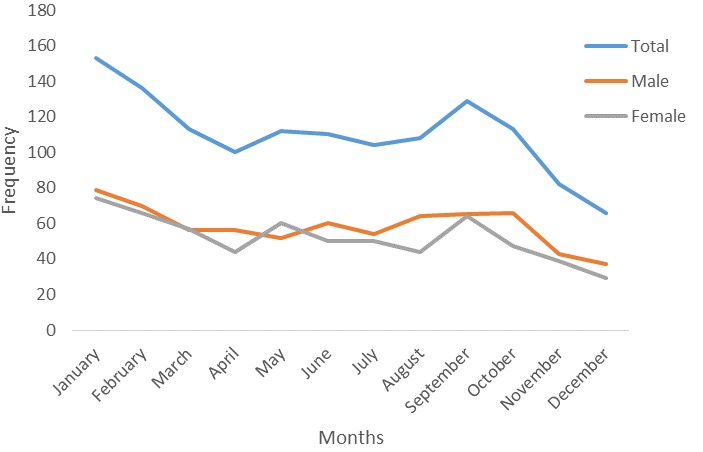
months of demise by sex, FMC Owo, 2006-2014

The total number of patients who died is shown in Figure 1. The peak number of death was in 2009 when 217 (16.4%) deaths were recorded. More males died yearly from 2006 till 2012 except in 2013 and 2014. In 2006, 16 (57.1%) males and 12 (42.9%) females died while in 2007, 81 (51.3%) males and 77 (48.7%) females died. In 2013, male deaths were 90 (46.4%) while female deaths were 104 (53.6%) and in 2014 male death were 77 (49%) while female deaths were 80 (51%). The peak number of death over the nine-year period was recorded in January 153 (11.5%) followed by February 135 (10.3%). The proportion of death that occurred monthly from 2006-2014 is as shown in Figure 2.

In children aged less than one year, low birth weight or premature birth were responsible for the deaths of 46% (75). Birth asphyxia caused the death of 64 children (39.3%). Among those one year old to 110 years HIV/AIDS related death occurred in 159 (15.2%) and bacterial infections 159 (15.3%) contributed more to death from communicable diseases, while cardiovascular diseases 168 (20.7%) and malignancy 86 (8.2%) were the leading cause of non-communicable disease death. Other leading causes of death are shown in Table 2. The highest number of deaths 210 (15.8%) occurred on Wednesdays. The median length of stay in hospital before death was 4 days (range: 0-277days). One hundred and forty four (10.9%) patients were not admitted. They were either brought in dead or died on the day they were brought to the hospital. Also, 16.4% died after spending one night on admission, those who died after more than 30 days on admission were 51 (3.8%). Table 3 shows the day of occurrence of death and overall length of stay.

**Table 2:** major causes of death among infants at Federal Medical Center, Owo, 2006-2014 (n=163)

Leading causes of death among < 1year	Frequency (n=163)	Percent
Low birth weight /Premature birth	75	46.0
Birth asphyxia	64	39.3
Neonataljaundice	19	11.7
Bacterial infection	5	3.1
**Leading causes of death among age ≥ 1-110**	(n=813)	
Cardiovascular Disease	168	20.7
Chronic Liver Disease	48	4.6
Renal Failure	55	5.2
Diabetes Mellitus	66	6.3
HIV/ AIDS related	159	15.2
Malignancy	86	8.2
Malaria	11	1.0
Tuberculosis	26	2.5
Bacterial Infection	160	15.3
Road Traffic Accident	34	3.2

**Table 3:** overall length of stay before death occurred, Federal Medical Center, Owo, 2006-2014

Length of stay before death (days)	Frequency	%
0	144	10.9
1	217	16.4
2-7	563	42.5
8-30	351	26.5
> 30	51	3.8
**Total**	**1,326**	**100.0**

**Figure 3:**
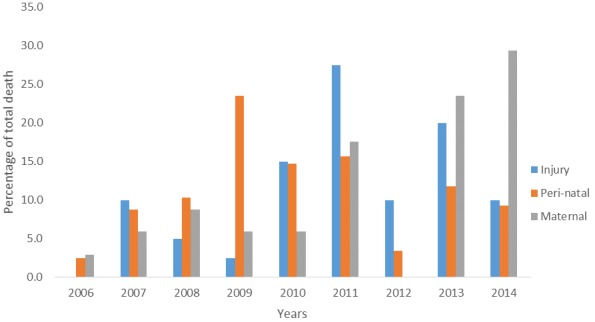
trend of all causes of death, FMC, Owo in five categories from 2006-2014

In 2011, injuries caused deaths in 11 (27.5%). In 2009 perinatal conditions lead to the death of 48 (23.5%). Death related to obstetric condition was at its peak in 2014, 10 (29.4%) were involved. Figure 3 shows the trend of all causes of death in categories from 2006-2014.

## Discussion

This secondary data analysis on mortality trends and its causes in a tertiary health facility and training institution showed that over a nine year period 1,326 deaths occurred. The median age at which death occurred was 46 years and was slightly higher in males. More deaths were recorded among males. The highest number of deaths was recorded in 2009. One in five deaths occurred among infants. The leading cause of death in the infants was low birth weight, asphyxia, neonatal jaundice and bacterial infection. Also, among those one year old and above 15.2% died of HIV and its complications. Injuries led to the death of 27.5% in 2011.

Mortality was highest in January. Patients usually avoid admission during the December festive period this could result in complications leading to death when they are seen after the festivity in January. Another study in Nigeria done at the Emergency Department of another hospital in Umuahia, in Southeast Nigeria reported that mortality was at its peak in July [9]. Though this study differ because it considered all mortality within the study period and it was not limited to only the Emergency Department. Many of the leading causes of death identified were preventable. HIV and bacterial infections contributed more to death from communicable diseases, while cardiovascular diseases and malignancy were the leading cause of non-communicable disease death. Non adherence to standard precaution has been shown to contribute to different types of communicable diseases like poor personal hygiene, and non-use of condoms correctly and consistently for sexual intercourse [10]. HIV was also a major cause of mortality similar to another tertiary health facility in Nigeria [11]. The major causes of non communicable diseases are lifestyle associated factors such as poor diet and lack of exercise. Diagnostic tools only help in early detection of some of these conditions.

However, inadequate use of preventive health care services and unavailability of tools for early detection of any deviation from normality are known causes of increase in the occurrence of non-communicable diseases. Low utilisation of maternal health services has been shown to be synonymous with poor birth outcome [12]. Other hospital-based mortality study in Nigeria have also reported similar causes of communicable and non-communicable diseases [13, 14]. Similar to findings of studies done in Obafemi Awolowo University Teaching Hospitals Complex, Ile - Ife, Osun State and Ogun State University Teaching Hospital in South West Nigeria more deaths occurred among males [15, 16]. Late health care seeking habit among men until complications sets in could be responsible [17]. One out of ten deaths occurred on the day the patients were brought to the hospital. Delay in seeking health care services and occurrence of death from road traffic crashes could lead to death of patients after few hours stay in the hospital [18, 19].

Injury was the leading cause of death from 2010 to 2013. The injuries were mainly from road traffic accidents. Globally road traffic injuries are currently ranked 9th among the causes of disease burden due todisability-adjusted life years (DALYs) lost. By 2020, road traffic injuries are projected to be the third largest cause of disabilities in the world [20].

This study has some limitations. Autopsy was often not done and when done, reports were not available. The absence of these reports to confirm cause of death may have led to over or underestimation of cause-specific mortality. Also, patients address was not included in the mortality data at the hospital therefore it was not possible to link the data with patient’s community. The findings from this study give the extent and the pattern of death in the catchment area of the hospital.

## Conclusion

The leading causes of mortality identified in this study are either preventable if detected early or treatable by seeking health care services promptly. HIV/AIDS related deaths are important causes of mortality in Nigeria. Also, infant mortality remains a large public health problem in Nigeria. Death from injury and road traffic crashes remain considerably high. The burden and prevalent forms of injuries in Nigeria need to be adequately characterized for appropriate intervention to be instituted. There is a pertinent need for road safety initiatives and road traffic injury surveillance to prevent injuries particularly those from road traffic accidents. Adoption and use of existing public health strategies and interventions are needed to reduce death from communicable diseases and death among infants.

### What is known about this topic

- Vital events registration is not accurately done in Nigeria;
- Mortality data is often unavailable;
- The leading causes of mortality in Nigeria are preventable.

### What this study adds

- Hospital mortality data could guide health decision making and intervention in Nigeria;
- Health facility-based data can provide useful information on the mortality and monitor trends in mortality of a population;
- There is a pertinent need for road safety initiatives and road traffic injury surveillance to prevent injuries particularly those from road traffic accident.

## Competing interests

The authors declare no competing interests.
